# Education and training for implementation science: our interest in manuscripts describing education and training materials

**DOI:** 10.1186/s13012-015-0326-x

**Published:** 2015-09-28

**Authors:** Sharon E. Straus, Anne Sales, Michel Wensing, Susan Michie, Bridie Kent, Robbie Foy

**Affiliations:** Li Ka Shing Knowledge Institute of St. Michael’s, University of Toronto, Toronto, Canada; VA Center for Clinical Management Research, VA Ann Arbor Healthcare System, Ann Arbor, MI USA; Faculty of Medicine, University of Michigan, Ann Arbor, MI USA; Radboud Institute for Health Sciences, Radboud University Medical Centre, Nijmegen, Netherlands; Centre for Behaviour Change, Department of Clinical, Educational and Health Psychology, University College London, London, UK; School of Nursing and Midwifery, Faculty of Health and Human Sciences, Plymouth University, Plymouth, UK; Leeds Institute of Health Sciences, University of Leeds, Leeds, UK

**Keywords:** Implementation science, Education, Training

## Abstract

Alongside the growth in interest in implementation science, there has been a marked increase in training programs, educational courses, degrees, and other offerings in implementation research and practice to meet the demand for this expertise. We believe that the science of capacity building has matured but that we can advance it further by shining light on excellent work in this area and by highlighting gaps for future research. At *Implementation Science*, we regularly receive manuscripts that describe or evaluate training materials, competencies, and competency development in implementation curricula. We are announcing a renewed interest in manuscripts in this area, with specifications described below.

## Editorial

Alongside the growth in interest in implementation science, there has been a marked increase in training programs, educational courses, degrees, and other offerings in implementation research and practice to meet the demand for this expertise [1–5]. At *Implementation Science*, we regularly receive manuscripts that describe or evaluate training materials, competencies, and competency development in implementation curricula. We have previously accepted a limited number of these. We are announcing a renewed interest in manuscripts in this area, subject to some specifications, which we describe below. We encourage interested authors to review our recent editorial describing the journal mission and scope, which provides additional details on the types of manuscripts we are seeking [6].

As noted in our earlier editorial, *Implementation Science* is focused on promoting the uptake of research findings into health care practice and health policy [6]. Articles that address building capacity in this area will be considered. Overall, we are most interested in manuscripts that describe the rigorous (i.e. using systematic, replicable, and valid methods) development and/or evaluation of educational and training interventions and resources to build capacity in the science or practice of evidence implementation in health care.

There are various target audiences for capacity building initiatives including those interested in becoming implementation researchers, researchers from other disciplines who want to gain necessary skills to facilitate appropriate dissemination and implementation of their own research, and decision makers including clinicians, undergraduate health care professional students, funders, policy makers, health care managers, and members of the public interested in the principles of evidence dissemination and implementation [5]. Given this wide range of learners, different types of educational and training activities are necessary to meet their needs. For example, comprehensive national training initiatives and graduate and postgraduate training opportunities (for individuals from a variety of backgrounds including those with clinical, business, and research training) have been developed to provide skills for those interested in becoming implementation researchers [2, 5]. Opportunities for researchers interested in developing skills to disseminate or implement their own research findings have also been developed including those by the NIH [1]. And, training for various decision makers is available to develop their skills in evidence implementation [4, 7] and to support others in its practice. Across these different target audiences, educators have taken various approaches from comprehensive training curricula [5] through to targeting specific competencies to focus training efforts [8]. Manuscripts that address any of these audiences will be considered.

Efforts have been made to identify the core competencies for various learners [5, 9, 10], highlighting how the implementation science field has advanced and how training must reflect these advances. In particular, with development in research methods and the building of the foundational science for implementation, training programs need to be flexible and create educational initiatives to meet these needs that evolve. For example, given the challenges in easily identifying relevant theory to inform the design of behaviour change interventions, workshops such as those by Michie and colleagues were created [11].

Finally, training initiatives have been provided in various formulations including in-person and online (both synchronous and asynchronous learning). Similarly, different dosages of training have been provided from brief one-off sessions to multiple sessions. This variability in offerings is helpful to meet the needs of different learners, and we anticipate receiving manuscripts addressing these different offerings.

### Scope and boundaries related to education and training manuscripts

#### Systematic reviews

We are interested in systematic reviews of capacity building in the science or practice of evidence implementation (Table 1). We will consider various review methodologies including scoping reviews, rapid reviews, and those that integrate qualitative and quantitative data.

**Table 1:** Scope of education and training manuscripts

Issue	Likely to be accepted	Likely to be rejected
Field of interest	Health care and population health	Anything else
Effectiveness studies	Evaluated the effectiveness of an intervention targeted to build capacity in the science or practice of implementation. Examples include studies evaluating implementation coaching, knowledge brokers, graduate curricula in implementation science, or continuing professional development courses in implementation	Evaluating the effectiveness of a patient education intervention
	We are interested in studies that use quantitative and/or qualitative methods to evaluate impact	
Process evaluations	Submitted with or following the report of intervention effectiveness as described above	Submitted without main intervention effectiveness paper
Training and educational intervention development reports	Prepared and submitted prior to the reporting of the effectiveness of the capacity building intervention	Description of a graduate course in theory without description of its development
		Description of a graduate course that is not going to be rigorously evaluated
	Developed using rigorous empirical and/or theoretical approaches	
Reports of measurement tools	Described the development and validation of a measurement tool to assess the impact of a capacity building initiative in the science and/or practice of implementation	Development of a measurement tool without validation or description of empirical methods used to develop it
Protocols	Described evaluation of a capacity building initiative in the science and/or practice of implementation	Protocol that has not been peer reviewed by a nationally recognised funding agency
	Peer reviewed by a nationally recognised research agency	
	Received ethics review board approval	
	Submitted prior to data cleaning or analysis	
Reports of development/evaluation of competencies	Described rigorous development of core competencies for implementation coach or scientist	No explicit methods used to develop core competencies
		No plan to evaluate competency-based intervention

#### Evaluations of capacity building interventions

We welcome studies that evaluate the effectiveness of an intervention targeted to build capacity in the science or practice of implementation. As outlined in our previous editorial, we expect studies that evaluate effectiveness to use rigorous and appropriate experimental or quasi-experimental designs [6]. Examples of potential studies include those evaluating implementation coaching, graduate curricula in implementation science, or continuing professional development courses in implementation practice. We are generally not interested in studies that evaluate the effectiveness of a patient education intervention as their goals are not aligned with capacity building in implementation. However, if the study evaluated a training program for patients to develop skills in the science or practice of implementation, it would be eligible. Similarly, we are less interested in programs that train health care professionals in the use of evidence-based practice as these initiatives are typically focused on enhancing research use at the individual patient-clinician level. We are also interested in studies that use qualitative or mixed methods for evaluating the capacity building intervention, although evaluations that focus purely on the experience of participants are of less interest to us. We are particularly interested in studies that describe capacity building initiatives that occur across more than one setting or country.

#### Process evaluations

We are keen to consider process evaluations of capacity building initiatives in the science or practice of implementation. In particular, we welcome studies that advance our understanding of the outcomes of effectiveness studies of capacity building initiatives. For example, the impact of context and type of learners on outcomes and the ‘dose’ and ‘formulation’ of the capacity building strategy are critical to advance knowledge in this area. We encourage authors to consider qualitative, quantitative, or mixed methods when developing their process evaluations, and we are interested in process evaluations that are submitted with or following the report of the intervention effectiveness. We are not interested in process evaluations that do not refer to the effectiveness of the capacity building initiative or that are submitted without the main intervention effectiveness paper.

#### Intervention development reports

We welcome manuscripts that describe the development of a capacity building initiative that use novel methods, and provide empirical or theoretical rationale for the content. These manuscripts should be submitted before the report of the effectiveness of the capacity building intervention is submitted to ensure the intervention was not modified after consideration of study outcomes. Similarly, we are not interested in descriptions of capacity building interventions that are not going to be rigorously evaluated. We require that authors include the course content as an appendix that we will make available on the Journal’s website.

#### Methods reports

We are interested in articles that advance methods for the study of capacity building. In particular, we welcome reports that describe the development and validation of measurement instruments to assess the impact of capacity building initiatives and that describe the development of competencies in implementation research or practice. We will typically reject articles that do not use explicit and rigorous methods for developing competencies or that do not plan to evaluate these competencies.

#### Protocols

We welcome protocols that describe the testing of a capacity building initiative in the research or practice of implementation. As noted in our recent editorial, we aim to publish protocols that have been peer reviewed by a nationally or internationally recognised research agency, that have received ethics approval, and that are submitted prior to data cleaning or analysis [6]. We encourage authors to refer to appropriate reporting guidelines to enhance transparency [12].

### Scope and boundaries related to content of education and training manuscripts

We welcome replications of research if they are accompanied by an appropriate rationale. We believe in treating effectiveness studies equally whether they report a positive, negative, or no effect on relevant outcomes. We are interested in studies that report outcomes relevant to capacity building in implementation research or practice, and these could include outcomes relevant to individuals, organisations, or the health system. At the individual level, these could include changes in attitudes, knowledge, skills, and behaviours. At the organisation level, these could include changes in processes of care or changes in culture, climate, or policy. At the health system level, changes in attitudes towards using research or actual research use in policy could be considered amongst others. We are interested in outcomes beyond ‘numbers of trainees’ that participated in educational events and their satisfaction with the events.

### Next steps

We are excited to witness the growth in interest in implementation science and in capacity building efforts to meet the demand. We believe that the science of capacity building has matured but that we can advance it further by shining light on excellent work in this area and by highlighting gaps for future research. We look forward to receiving manuscripts that reflect innovative work in this field and also invite authors to provide feedback on our approach. We will endeavour to continue to revisit our scope, using reflection on the field and input from our readers.
